# Navigating Through Electronic Health Records: Survey Study on Medical Students’ Perspectives in General and With Regard to a Specific Training

**DOI:** 10.2196/12648

**Published:** 2019-11-12

**Authors:** Anne Herrmann-Werner, Martin Holderried, Teresa Loda, Nisar Malek, Stephan Zipfel, Friederike Holderried

**Affiliations:** 1 Department of Psychosomatic Medicine and Psychotherapy Internal Medicine University Hospital Tübingen Tübingen Germany; 2 Process and Quality Management Department of Medical Structure University Hospital Tübingen Tübingen Germany; 3 Department of Gastroenterology, Hepatology and Infectious Diseases Internal Medicine University Hospital Tübingen Tübingen Germany

**Keywords:** medical students, electronic health records, eHealth, simulation

## Abstract

**Background:**

An electronic health record (EHR) is the state-of-the-art method for ensuring all data concerning a given patient are up to date for use by multidisciplinary hospital teams. Therefore, medical students need to be trained to use health information technologies within this environment from the early stages of their education.

**Objective:**

As little is known about the effects of specific training within the medical curriculum, this study aimed to develop a course module and evaluate it to offer best practice teaching for today’s students. Moreover, we looked at the acceptance of new technologies such as EHRs.

**Methods:**

Fifth-year medical students (N=104) at the University of Tübingen took part in a standardized two-day training procedure about the advantages and risks of EHR use. After the training, students performed their own EHR entries on hypothetical patient cases in a safe practice environment. In addition, questionnaires—standardized and with open-ended questions—were administered to assess students’ experiences with a new teaching module, a newly developed EHR simulator, the acceptance of the health technology, and their attitudes toward it before and after training.

**Results:**

After the teaching, students rated the benefit of EHR training for medical knowledge significantly higher than before the session (mean 3.74, SD 1.05). However, they also had doubts about the long-term benefit of EHRs for multidisciplinary coworking after training (mean 1.96, SD 0.65). The special training with simulation software was rated as helpful for preparing students (88/102, 86.2%), but they still did not feel safe in all aspects of EHR.

**Conclusions:**

A specific simulated training on using EHRs helped students improve their knowledge and become more aware of the risks and challenges of such a system. Overall, students welcomed the new training module and supported the integration of EHR teaching into the medical curriculum. Further studies are needed to optimize training modules and make use of long-term feedback opportunities a simulated system offers.

## Introduction

Electronic health records (EHRs) comprise health information of a patient showing clinical data collected from all professionals involved in the patient’s care, including nurses, doctors, therapists, laboratories, and external specialists [1].

Besides the immediate integration of a wealth of clinical data and examination results, implementing the usage of EHR provides numerous benefits, including increased adherence to guidelines in preventive care, decreased paperwork for providers, improvement in overall quality, efficiency of patient care [2], reduction of errors [3,4], enhanced monitoring of drug therapy [4], better daily workflow management [5], easy access of clinical data, legibility of notes, improved problem and medication lists, and better preventive care documentation. Challenges and risks, however, have been reported regarding heightened susceptibility to automation bias, decreased quality of notes because of copying and pasting [6,7], alert fatigue (desensitization) [8], disruption of the patient-physician relationship [9], mismatch of human and machine workflow models, and productivity loss potentially caused by EHR usability issues [2].

However, despite all the current knowledge of the benefits and risks of use of EHR and other technology, there has not been much research on the acceptance of new health technology systems such as EHR particularly among students. Students seem to be generally positive and more receptive to new technologies than more experienced health care providers [4,10]. The acceptance of health technology is mainly influenced by 2 underlying factors: the devices’ *perceived ease of use* and *perceived usefulness* [11,12]. More perceived ease of use and higher usefulness might also underlie the findings of Tierney et al [10], with medical students as *digital natives* being closer to technology systems. However, medical students—despite their high exposure to and experience with electronic media—still need specific training in electronic health care systems as they rate their ability to use such clinical information systems as rather low [3,13-16]. The need for training is also mirrored by the fact that accreditation bodies and national catalogs of learning objectives expect medical graduates to be able to communicate clearly orally and in writing, including the documentation process in medical charts coining it a core competency [15,17-19]. However, so far, not enough clarity has emerged as to how and when such training in EHR usage should be integrated into the medical curriculum and which specific competencies should be reached [15,20-22]. In addition, Berndt and Fischer in their recent review [20] concluded that the growing use of EHR “for medical education, [...] poses many new challenges for teaching and learning (e.g., teaching of new data management skills; new roles and responsibilities for students and teachers) which have hardly been addressed.” Previous studies have shown that training in the implementation process of EHR in general is useful [2], training in EHR has specifically improved communication when using the EHR [23], and training in the usage of EHR should already be in the focus of medical education fairly early on [9,15]. This also takes into account that most errors in EHR usage come down to issues concerning adequate training, well-prepared implementation, and the possibility of getting accustomed to the system [24,25].

Also requiring attention in line with these considerations are the technical, ethical, and legal points accompanying such training. Before digitalization, students could simply walk into the nurses’ station and pick up the paper chart [15]. With EHR, the procedure is quite different as students now need individual login data, and unfortunately, a lot of medical schools deny their students permission to document EHR live, which lessens the potential benefit EHR can have in medical education and might lead to information loss within health care teams [26-28]. Despite the widespread usage of EHR in clinical practice in Germany and elsewhere, surveys show that medical students are often not allowed to make use of its full potential [3,26,29,30]. Simulated training environments offer a safe solution to this issue and are well accepted by students but are so far only rarely used [24,31-33]. However, clear rules of responsibility have to be defined when students are working with live EHR, particularly when considering the complicated general legal regulations in the European Union and the German system [34,35].

In summary, students need access to EHR to become knowledgeable and skilled in its use and to improve their understanding of system-based practice, because future medical practice environments will likely include the use of EHR. As students use EHR regardless of prior preparation, the need for training guidelines definitely exists [3,15,20]. Just as medical schools currently teach proper documentation as part of good clinical care in a paper-based world, they should be similarly obligated to teach students proper use of an EHR in an increasingly electronic world [26]. Atwater et al [9] concluded that “Best practices and strategies for teaching medical trainees in the setting of EHR have not been identified or widely shared with the medical education community.” Thus, in this study, we aimed to develop a course module and evaluate it to offer a best practice teaching example.

## Methods

### Study Design and Participants

This longitudinal study took place at the Medical Faculty of the University of Tübingen in summer term 2018. A paper-based questionnaire was administered before and after the teaching session on EHR. Fifth-year medical students were recruited within their regular seminar in internal medicine. Participation in the EHR training was mandatory. However, participation in this study was on a voluntary basis. Out of 171 students, 116 (response rate 68.8%) participated in the study.

### Test System

Teaching was conducted using a specially designed test system that exactly mirrored the EHR software program *Meona* (Meona GmbH, Freiburg, Germany) used in the clinical service at the University Hospital of Tübingen. It was created with 2 imaginary wards (internal medicine and surgery), allowing the virtual accommodation of up to 28 patients. Patient cases were developed by clinical experts in internal medicine before the teaching began. The cases were either simply created as plain characters or entered with a full medical history and doctor’s orders depending on the respective purpose. As there was no link to the actual EHR (Meona) version in clinical use, it provided a safe training environment without any implications for real patients. At the same time, however, the students were able to practice with a perfectly realistic copy of the original EHR system. The system was created and supported by the Information Technology (IT) Department of the University Hospital, which also maintains the actual clinical version. Once every 24 hours, it had to be updated after which one could either use the new blank version or upload the screenshot from before the update.

### Teaching

The teaching course on EHR was held as a full-day intensive training over 2 consecutive days (6 hours per day). Before the actual teaching on day 1, students had to fill in the first questionnaire (T_0_). Afterward, teaching started with a lecture on the general advantages, disadvantages, and pitfalls of EHR as well as a specific training on how to use the Meona system. As EHR count as a medical device in Germany demanding formalized training, part of the teaching was a standardized video on how to use the EHR system. This was followed by an interactive class including a lecture on how to perform a chart review and common medical errors to avoid. After lunchbreak, students were shown specific procedures within the EHR system (eg, tasks when admitting or discharging a patient) and had ample time to practice with a fictive patient, who was created as a new admission, with the student being asked to enter all the necessary information into the system and make orders accordingly. Day 1 ended with a wrap-up discussion exchanging experience using the EHR. On day 2, teaching started with a short refresher course on main points from the day before. Afterward, students were given specifically designed patient cases to perform a chart review. The cases covered typical patients seen in internal medicine (eg, complicated diabetes and gastrointestinal bleeding). Students first had to work on their own; this was followed by an interactive discussion including medical and technical issues. At the end of day 2, students filled in the second questionnaire (T_1_).

Teaching was conducted by 2 experienced clinicians who each held a certificate as an official Meona instructor as well as a Master’s Degree in Medical Education.

### Questionnaire

We developed a questionnaire based on literature-derived common themes in EHR use and adapted from prior questionnaires in use [9,15,36]. The questionnaire had undergone cognitive pretesting using the method of *think aloud*, where the subject concurrently verbalizes thoughts when answering a questionnaire [37,38]. Consequently, minor adaptions to the questionnaire were made, and it was administered pre teaching (T_0_) and post teaching (T_1_) to allow for comparisons. The questionnaire can be obtained upon request. Students provided basic sociodemographic data (eg, age, gender, and semester), former training data, IT/electronic health–related data (eg, possession of devices and usage of the internet for health topics), and information regarding their prior experience with traditional chart reviews as well as EHR. In addition, they rated the general potential of EHR as well as the specific benefit for different professional groups (students, physicians, nurses, patients, and other professional groups) and their collaboration. Students also rated the teaching and the test system used. Table 1 provides an overview on the items used.

**Table 1:** Overview of outcomes and their corresponding measurement of the questionnaire.

Outcome	Item	Number of items
Sociodemographics	Gender, age, and response rate	3
Previous experience with electronic devices (eg, mobile phones, personal computer, and laptop)	Yes/no	6
Previous experience with EHR^a^ (participation, contribution, and contact)	Yes/no	5
Benefit for different professions	Likert scale from 0 to 5 (“not at all” to “completely”)	6
Concerns and inhibitions	Likert scale from 0 to 3 (“not at all” to “completely”)	3
Evaluation of the test system	Likert scale from 0 to 3 (“not at all” to “completely”)	6
Evaluation of the teaching module	Likert scale from 0 to 3 (“not at all” to “completely”)	5
Students’ experiences with EHR	Likert scale from 0 to 3 (“not at all” to “completely”)	6

^a^EHR: electronic health record.

### Data Analysis

Data analysis was performed using SPSS version 24. For statistical analysis, frequencies, means, and associated SDs were calculated for different items of the questionnaire. Data were normally distributed as tested by the Kolmogorov-Smirnov test. *T* tests for 2-paired samples were conducted to allow comparisons of pre teaching and post teaching. For further comparison, analyses of variance were conducted. Here, the level of significance was *P*<.05. For the comparison of pre teaching and post teaching, data were included only when the students filled in both questionnaires. Furthermore, we considered the cumulative frequencies in percentages for several items such as prior usage of EHR. Here, questionnaires of all 116 students taking part in the study were included, and frequencies were calculated proportionately for each item. At the end of the study, 104 out of 116 students had returned the complete pre- and postquestionnaires and could be included in the analyses of comparisons. Again, on the singe-item level, frequencies were calculated proportionately. The absolute numbers might differ slightly from 116 or 104 students because of missing data.

### Ethics

The Ethics Committee of Tübingen Medical Faculty (#260/2016BO2) approved this study.

## Results

### Sociodemographics

A total of 116 students participated in the study, and 104 students returned the completed pre- and postquestionnaires and showed up for both appointments of the study. Moreover, 59 (56.7) students were female and 45 (43.3) were male. Their mean age was 25.6 (SD 3.0) years.

### Previous Experience With Electronic Devices

Nearly all the students (103/113, 91.1%) had a mobile phone, 111 (98.2%) had a personal computer with internet connection, and 79 (69.9%) owned a tablet. Out of 112 students, 76 (67.8%) stated owning all 3 devices, and 108 (96.4%) students rated the internet as *rather important* or *important* for their daily lives. Students checked their private emails every day, which was significantly more often than their professional ones (*F*_1,4_=38.04; *P*<.001).

### Previous Experience With Electronic Health Records

Out of 104 students, 67 (64.4%) had already participated in a chart review in general (paper or EHR). However, out of these, only 18 (27%) students had actively contributed to one. Mostly, the chart review was part of their mandatory clinical placements. In addition, 66 out of 101 (65.3%) students already had contact with an EHR system, with proportionally the largest group (36/47, 77% students) having watched someone else using it. Finally, 99 out of 103 students (96.1%) had thus far no formal training in EHR.

### Benefit for Different Professions

The students’ judgment of the relative benefit of EHR for medical professionals did not vary significantly between T_0_ and T_1_ regardless of the group (see Table 2). In addition, students rated the benefit of EHR significantly higher for doctors and nurses than for any other professions both before and after training (pre teaching—benefit doctors, mean 4.11; nurses, mean 3.90; therapists, mean 3.67; patients, mean 3.10; medical students, mean 3.55; *P*<.001 for doctors and nurses compared with all other professions—and post teaching—benefit doctors, mean 3.96; nurses, mean 3.82; therapists, mean 3.68; patients, mean 3.29; medical students, mean 3.68; *P*<.001 for doctors and *P*=.03 for nurses compared with all other professions). Analyzed in detail, students rated the benefit of EHR for their medical knowledge significantly higher after the teaching session (Table 2).

**Table 2:** Ratings of benefits, concerns, and inhibitions of electronic health record.

Item	Pre teaching^a^, mean (SD)	Post teaching^a^, mean (SD)	T_0_–T_1_ comparison	
			*t* test (*df*)	*P* value
Benefit for doctors	4.11 (0.88)	3.96 (0.93)	−0.84 (100)	.40
Benefit for nursing stuff	3.90 (0.98)	3.82 (0.99)	0.92 (99)	.36
Benefit for physiotherapist or speech therapist	3.67 (1.05)	3.68 (0.95)	−0.12 (97)	.90
Benefit for patients	3.10 (1.31)	3.29 (1.32)	−1.52 (97)	.13
Benefit for students	3.55 (1.18)	3.68 (1.08)	−1.16 (97)	.25
Benefit for students’ medical knowledge	2.96 (1.27)	3.29 (1.11)	−2.86 (98)	.005
General concerns	0.63 (0.89)	0.69 (0.86)	−0.58 (101)	.56
Inhibitions	0.40 (0.76)	0.38 (0.77)	0.26 (100)	.80
Potential as a collaboration tool	3.18 (0.87)	3.01 (0.88)	1.99 (99)	.049

^a^Agreement (“0” = “not at all” to “5” = “completely”).

### Concerns and Inhibitions

There was no significant difference before and after training regarding concerns and inhibitions related to EHR use. However, students evaluated EHR’s potential long-term benefit as a collaboration tool in the multiprofessional health care team to be significantly lower at T_1_ compared with T_0_. Table 2 provides further details.

### Evaluation of the Test System

The most frequently mentioned positive aspects were the protected and safe environment (29/58 students, 50%) in which to practice as well as the general benefits of an EHR system such as drug interaction warnings. EHR needs a substantial amount of training with proper facilities (16/70 students, 23%) and the fear that other hospitals might have different systems (2/70, 3%) for which they would then not be prepared were some of the critical issues mentioned by the students. In addition, students pointed out that the training system still had some technical difficulties (23/70, 33%; eg, no immediate connection to current treatment guidelines and inappropriate date of birth of the created patients). When presented with a list of areas where support in the future would be needed most, issues concerning active processes such as *change the patient’s medication* (17/104 students, 16%) and *confident navigation through the system* (28/104 students, 27%) were among the most frequent answers.

### Evaluation of the Teaching Module

Out of 102 students, 88 (86.3%) stated that the teaching prepared them in a *rather good* or *good* way for later usage of EHR. When asked for which area they felt best prepared specifically (eg, navigation, patient admission, placing orders, and changing medication), there was a significant difference among the subthemes (*F*_6,64_=3.59; *P*=.002), with students feeling best prepared for reading and understanding the current medication scheme (mean 2.25, SD 0.66) and worst prepared for navigation through the EHR (mean 1.96, SD 0.65). Out of 103 students, 44 (42.7%) would have liked to have the teaching video in a web-based version as well, with another 24 students (23.3%) agreeing that it might be helpful to have such an additional option. However, they also generally appreciated the presence of a real teacher, as 74 out of 103 students (71.8%) stated that a web-based teaching program alone would *not* or *possibly not* be sufficient to reach the desired competencies and understanding.

**Figure 1:**
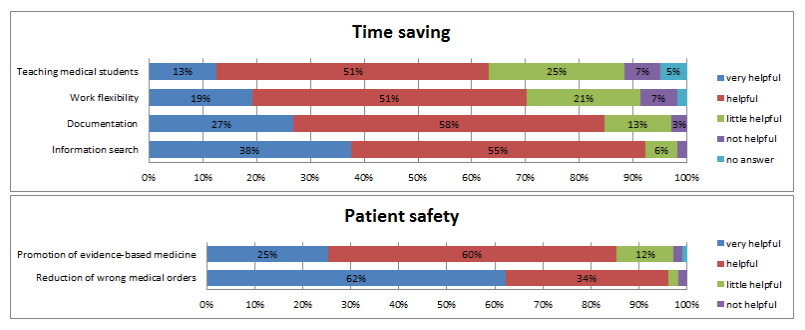
Percentage of students (N=104) who found the electronic health record very helpful, helpful, little helpful, and not helpful, respectively, with regard to time saving and patient safety aspects. Values less than 3% are not marked on the graph to improve readability.

After the teaching, the students felt rather motivated to work with EHR in the future (mean 3.74, SD 1.05) and considered EHR as a useful tool in clinical practice (mean 3.7, SD 0.04). Looking closer at different aspects of time saving and patient safety when using EHR, EHRs were mostly considered as a *very helpful* or *helpful* tool in later work in hospitals by our students. For the advantages and details they identified, see Figure 1.

In addition, 82 out of 104 students (78.8%) considered the system’s offer of templates (eg, normal findings on physical examination and electrocardiography) *helpful* or *very helpful*. Reasons for finding it only *somewhat helpful* or not helpful were *producing data waste*, *limitation of expressions*, and *standard formulations are known by heart anyway*. Accordingly, 98 out of 104 students (94.2%) considered the integrated support system of EHR (eg, immediate warnings about drug interactions) *helpful* or *very helpful*. The 6 students considering the system *not helpful* or *only somewhat helpful* were most critical on the following points: *giving a false sense of security* (n=3), *danger of not thinking critically on one’s own* (n=1), and *limited flexibility through forced adherence to guidelines* (n=1). However, the problem of alert fatigue, as mentioned in the Introduction, was not reported by our students. Our medical students seemed unsure about how to judge the potential problem of *copy and paste*—also one of the main risks and pitfalls of EHR—rating it mean 2.3 (SD 1.5) on the abovementioned scale. Of the 48 students being more reserved toward the copy and paste possibility, the majority mentioned worries along the issue of *blind take-over of information/no cross-checking/no reflection on (potentially wrong) diagnoses* (n=41). Only 3 students were concerned about the potential issue of *loss of quality/reduced doctor-patient interaction*.

## Discussion

### Summary

This study looked at undergraduate medical students’ perspectives toward EHR in general and with a specific focus on a particularly designed teaching module. Teaching included a formalized introduction to EHR accounting for legal demands as well as plenty of contextual training applying medical knowledge in the electronic system.

### Perspectives on Electronic Health Records

In general, the students in this study had a positive attitude toward EHR usage, which is generally in line with previous findings [4,10]. Interestingly, participants rated the benefit of EHR usage significantly higher for doctors and nurses than for other health care professionals or their patients at both measurements. This might be because of the fact that medical students still mostly see those 2 professions in action of patient care. Interestingly, students in this study rated the potential benefits for coworking in a multiprofessional team significantly lower after their teaching sessions. This seems counterintuitive as common access to medical charts should foster team collaboration, although caveats have been described in literature with current systems yet lagging the full potential [39-41]. The students’ reservations might be explained by the fact that through specific training, they become aware not only of the advantages but also of the shortcomings of EHR usage, as already mentioned in the Introduction, enabling them to evaluate clinical information systems more critically. In addition, they have not been using them in real clinical practice. Therefore, they can only imagine and anticipate or remember complex interactions they have been observing in clinical internships where frequent difficulties and problems with EHR are discussed more prominently than effectively working examples in team interactions. The possible influence of EHR on the patient-physician relationship was not an issue for our students. This is in line with literature showing effective patient-centered interactions despite usage of EHR in the encounters [42,43].

Although clinical decision aids integrated into EHR offer great learning opportunities, there is a danger of *alert fatigue* in users [8,44]. Students in this study did not show such concerns. We assume that this phenomenon might not be prominent in students, who have not been using the system frequently so far but is more of an issue for experienced system users working with EHR on a daily basis. However, it seems crucial to create an awareness of this issue early on. This also accounts for possible negative implications of the *copy&paste* phenomenon. Our medical students were unsure how to rate this issue, but those concerned named well-known reasons in the clinical context [6,7]. So far, literature has not shown any negative educational consequences of copy and paste (eg, impaired critical thinking and reduced self-directed learning). However, there is certainly the need for more standardized examinations on that matter [44-46].

### Teaching

In this study, students accepted the new technology well and felt highly motivated to use EHR. They all represented the generation of digital natives—as reflected in their possession and usage of technical devices and the internet and students thus might be especially receptive to the use of new technologies [10]. However, this might not be transferable to an adequate professional use, and despite being digital natives, students do need specific training in technical devices in the health care context [3,13,14,16]. As documentation is a core competency that graduates should show from day 1 of their clinical work, the need for specific training in the usage of EHR is thus undisputable [15,17,19]. There are even demands of whole longitudinal curricula on this issue [15]. This enables several levels of reality: starting with theoretical input in the early years and proceeding to simulated scenarios as well as a structured integration of live EHR use in clinical placements. However, reality does look different: students usually are not officially allowed to document in EHR or sometimes do so without proper training in the systems beforehand [3,26,29,30]. The students in this study also reported mostly just having watched someone using an EHR. However, some students had documented on their own but without training, which poses a legal problem in Germany as EHR count as a medical device is not allowed to be used without a formalized introductory teaching beforehand. Directors of medical schools should be aware of this potentially dangerous issue.

Although the students evaluated their training course positively, it does not seem to have been thorough enough, as students still did not feel safe when navigating through the EHR afterward. This uncertainty might have resulted from students focusing on the medical information and casework, prioritizing this part of the task over organizational and structural learning objectives of this class. This was reflected in the in-class discussions where students’ questions mainly concerned medical issues, and EHR seemed to be merely a means for that purpose. A lack of EHR navigating skills is also what Morrow et al [23] discuss when finding that after training, students had significantly better communication skills within the EHR tool but did not show satisfying navigation skills such as finding previous data or creating trend graphs. It may be necessary to separate medical content from technical information or at least specifically stress the importance of structural skills [20].

### Simulated Electronic Health Record System

When looking at students’ experiences with the new EHR software program (Meona) in general, the feedback was positive. Overall, the students appreciated the features they would also encounter in the live version, although they were also aware of the technical difficulties still present in the newly developed copy of the actual Meona. We want to draw attention to some of these, as we consider this as helpful for other medical schools planning to develop an EHR simulator. When creating a teaching version of an in-use EHR, it is important to keep in mind that the system needs constant updating. In our case, this meant reinstalling the initial version to delete the entries of one student group before the next one works with the program. However, this means that when you have admitted the patient in April and constantly back up to this initial version, the students who have their training class in June are supposed to work with patients who have been on the ward for 2 months with nothing having been done up to this point. In the whole process, it is also crucial to involve IT [47]. This accounts for making them familiar with the content of your teaching before they start to program the virtual patients. It does not foster the degree of felt reality when students work on 19-year-old patients who have been in and out of hospital for the past 20 years because of their poorly controlled diabetes. When creating such a system, it is also important to predefine who will be responsible for tasks, who has the administrator’s rights, and when the program is to be updated or reloaded so that you have a secured environment [47]. During our first term of teaching EHR, not having clarified all these issues, we more than once had to manually reenter all patient data because IT made an update without a screenshot first. In addition, in the beginning, we were unable to change minor issues ourselves as we did not have the rights to do so. Thus, it might be helpful to have key users with limited administrative rights who can customize the system accordingly, such as only updating it during semester break.

Simulated systems are created to prepare for reality. There is ample literature regarding the rights of medical students in live EHR systems [3,48]. When using real systems, one has to find the balance between allowing students to be part of the team with the same duties and ownership as other team members on the one hand [3], whereas, on the other hand, taking into account legal issues of responsibility that might exceed a student’s capability level and will need to be reviewed [49,50]. By choosing a mirrored version of the actual EHR system used in our hospital, all students in the class automatically got the training necessary to be allowed to work with the live electronic chart. As a consequence of this teaching, the Medical Faculty of the University of Tübingen together with the Quality Management Department of the University Hospital defined and implemented those rights for all students in their final year to ensure quality of care and reproducibility of the clinical documentation within the EHR system. One key element of this process was to show students’ entries color coded as *preliminary documentation* that has to be checked and confirmed by a fully trained physician before release, although this has been shown to be a source of concern among deans of medical schools [30,45]. Thanks to such provisions, students could start using EHR immediately the day they entered their final practical year without endangering patient safety.

### Limitations

The study has several limitations. First, we only looked at medical students at one semester and one faculty, which limits generalizability. In addition, the training class was relatively short, being an intensive course over 2 days; thus, some of the results might be not representative enough. Finally, we did not look at transfer into the clinical environment, thus not being able to say if the students’ self-ratings would hold up in the actual context of use.

Despite these limitations, we strongly believe that our study delivers valuable insight into aspects of consideration when planning and implementing a teaching class on EHR into the medical curriculum.

### Conclusions

Overall, the class showed several advantages, and the training was regarded as helpful. However, it might have been more helpful to separate medical content from the technical aspects to reduce cognitive overload or have at least more teaching time longitudinally, as already practiced in some medical schools [20,51]. Future development could include assigning person-specific logins to track individual progress. In addition, the potential of interprofessional as well as nationwide or even worldwide web-based learning opportunities should be considered [52].

## Abbreviations

EHR: electronic health record
IT: information technology
